# The effect of glycopyrrolate vs. atropine in combination with neostigmine on cardiovascular system for reversal of residual neuromuscular blockade in the elderly: a randomized controlled trial

**DOI:** 10.1186/s12871-024-02512-x

**Published:** 2024-04-01

**Authors:** Yanping Wang, Liyuan Ren, Yanshuang Li, Yinhui Zhou, Jianjun Yang

**Affiliations:** Department of Anesthesiology, Pain and Perioperative Medicine, The First Affiliated Hospital of Zhengzhou University, No.1 East Jianshe Road, 450052 Zhengzhou, China

**Keywords:** Glycopyrrolate, Atropine, Neostigmine, Cardiovascular system, Elderly

## Abstract

**Background:**

Glycopyrrolate-neostigmine (G/N) for reversing neuromuscular blockade (NMB) causes fewer changes in heart rate (HR) than atropine-neostigmine (A/N). This advantage may be especially beneficial for elderly patients. Therefore, this study aimed to compare the cardiovascular effects of G/N and A/N for the reversal of NMB in elderly patients.

**Methods:**

Elderly patients aged 65–80 years who were scheduled for elective non-cardiac surgery under general anesthesia were randomly assigned to the glycopyrrolate group (group G) or the atropine group (group A). Following the last administration of muscle relaxants for more than 30 min, group G received 4 ug/kg glycopyrrolate and 20 ug/kg neostigmine, while group A received 10 ug/kg atropine and 20 ug/kg neostigmine. HR, mean arterial pressure (MAP), and ST segment in lead II (ST-II) were measured 1 min before administration and 1–15 min after administration.

**Results:**

HR was significantly lower in group G compared to group A at 2–8 min after administration (*P* < 0.05). MAP was significantly lower in group G compared to group A at 1–4 min after administration (*P* < 0.05). ST-II was significantly depressed in group A compared to group G at 2, 3, 4, 5, 6, 7, 8, 9, 11, 13, 14, and 15 min after administration (*P* < 0.05).

**Conclusions:**

In comparison to A/N, G/N for reversing residual NMB in the elderly has a more stable HR, MAP, and ST-II within 15 min after administration.

## Introduction

Neuromuscular blocking drugs can assist in tracheal intubation, mechanical ventilation, and improving surgical conditions during general anesthesia [1, 2]. However, it causes incomplete recovery from neuromuscular blockade (NMB), which is strongly linked to adverse postoperative respiratory events [3–5]. Muscle relaxation antagonists should be routinely used to reverse residual NMB in patients undergoing general anesthesia [6, 7]. Currently, muscle relaxation antagonists used in clinical practice include glycopyrrolate-neostigmine (G/N), atropine-neostigmine (A/N), and sugammadex.

Sugammadex can rapidly and effectively reverse deep NMB than neostigmine, with fewer postoperative pulmonary complications [8–10]. However, its higher cost and the fact that it can only selectively antagonize aminosteroid (but not benzylisoquinolinium) relaxants affect its prevalent use in clinical practice. A/N for reversing residual NMB causes significant fluctuations in heart rate (HR), which have no negative consequences for most young patients. However, in elderly patients with poor cardiovascular reserve function and mostly coexisting cardiovascular disease, significant HR fluctuations may induce unwanted cardiovascular events [11–13].

The pharmacokinetics of glycopyrrolate are synchronous with those of neostigmine [14, 15]. Several studies have found that G/N results in less change in HR than A/N [16–19]. This advantage may be especially beneficial for the elderly in reducing cardiovascular responses and the occurrence of cardiovascular events such as myocardial ischemia and arrhythmias. However, the cardiovascular effects of G/N in elderly patients have not yet been adequately investigated. Therefore, the purpose of the current study was to focus on elderly patients at increased risk of cardiovascular complications and compare the cardiovascular effects of G/N and A/N for the reversal of NMB. We hypothesized that G/N is more stable for HR, mean arterial pressure (MAP), and ST segment in lead II (ST-II) than A/N within 15 min after administration.

## Materials and methods

### Study design and ethics

This prospective, randomized, double-blind study was conducted in patients undergoing elective non-cardiac surgery at the Anesthesiology and Operation Center of the First Affiliated Hospital of Zhengzhou University. The study was approved by the Scientific Research and Clinical Trial Ethics Committee of the First Affiliated Hospital of Zhengzhou University (Ref: 2022-KY-0763-002, 31/05/2022), and registered in the Chinese Clinical Trials Registry (clinical trial registration No: ChiCTR2200061576, 29/06/2022). Written informed consent was obtained from all participants. The trial report complies with the Consolidated Standards of Reporting Trials (CONSORT) checklist.

### Participants

We recruited elderly patients scheduled for elective non-cardiac surgery under general anesthesia from June 2022 to September 2022. Inclusion criteria were as follows: (1) aged 65 to 80, male or female; (2) American Society of Anesthesiologists (ASA) class I or II; (3) 18 ≤ body mass index (BMI) ≤ 28; (4) the expected operation time < 4 h; (5) planned use of neostigmine to antagonize residual NMB; (6) voluntary participation in this trial and signed the informed consent.

Exclusion criteria were as follows: (1) allergies to anticholinergic drugs and their components; (2) respiratory, circulatory, and digestive system diseases that affect HR; (3) use of medications affecting the cholinergic system; (4) poor communication skills.

### Outcomes

The primary outcomes were HR at 1 min before administration and 1–15 min after administration. Secondary outcomes included: (1) MAP at 1 min before administration and 1–15 min after administration; (2) ST segment changes in lead II (changes in ST-II) at 1–15 min after administration.

### Data collection

Noninvasive blood pressure (NBP) was measured by an upper arm cuff at an automatic interval of 3 min intraoperatively, which was then adjusted to a 1-minute interval before administration of the test drug until 15 min after administration. HR, MAP, and ST-II were recorded automatically every 1 min from 1 min before (the baseline) until 15 min after administration by a multifunction monitor. After the observation, data were collected via electronic recording of the same monitor.

### Randomization and blinding

Using a computer-generated randomization table, patients were randomly assigned in a 1:1 ratio to either the glycopyrrolate group (group G) or the atropine group (group A). Group assignments were secured in a sequentially numbered, sealed opaque envelope, which was then handed to a nurse who was not involved in the study. Test drugs were prepared in identical 10 mL syringes by the same nurse. The anesthesiologists involved in anesthesia management and postoperative follow-up were blinded to the group assignments.

### Anesthetic and surgical management

Once in the operating room, all subjects underwent standard monitoring, including electrocardiogram (ECG), NBP, pulse oxygen saturation (Sp0_2_), and bispectral index (BIS). After inducing general anesthesia with alfentanil (0.03–0.08 mg/kg), etomidate (0.2–0.3 mg/kg), and cisatracurium (0.15 mg/kg), tracheal intubation was performed and mechanical ventilation initiated. The appropriate depth of anesthesia (BIS value 40–60) was maintained by micropump infusion of propofol (2–4 mg/kg/h), remifentanil (0.05–0.15 ug/kg/min), and 0.5–2 vol% sevoflurane/oxygen/air mix inhalation. Intraoperative blood pressure was kept within 20% of baseline, and HR was maintained at 45–90 beats per min (bpm). 1/3 of the induction dose of cisatracurium was added intraoperatively as needed. There were no additional muscle relaxants administered for at least 30 min before the procedure ended. At the end of the surgical procedure, the administration of sevoflurane inhalation, propofol, and remifentanil pumping was discontinued.

Following the last administration of muscle relaxants for more than 30 min, antagonizing residual NMB was performed. Group G received an intravenous injection of 4 ug/kg glycopyrrolate and 20 ug/kg neostigmine, whereas group A received an intravenous injection of 10 ug/kg atropine and 20 ug/kg neostigmine. Vital signs were closely monitored, and the tracheal tube was removed if respiratory recovery was satisfactory and extubation criteria were met. Intravenous 5 ug/kg atropine was given as treatment if the patient’s HR fell below 45 bpm during the observation period.

### Sample size calculation and statistical analysis

In this study, the first 20 patients enrolled were used to calculate the sample size by power analysis. The primary variable studied was HR at different time points in the two groups, which was analyzed by repeated measurements ANOVA using SPSS 26.0, and the bias η² of the time × group interaction was 0.251. The effect size was calculated as 0.58 by Gpower 3.1, which is considered a relatively significant difference. To achieve a power of 0.99 at a type 1 error of 0.05 based on the repeated measurements ANOVA, a sample size of 27 in each group was required. To account for dropouts, 34 patients were finally included in each group.

Statistical analysis was performed using SPSS 26.0. The normal distribution of the variables was examined using the Shapiro-Wilk test. Normally distributed measures were expressed as mean ± standard deviation (x ± s), and t-test for independent samples was used for comparison between groups. Non-normally distributed measures were reported as median (M) and interquartile range (IQR), and Mann-Whitney U test was used for comparison between groups. Count data were expressed as cases (%), and χ2 test or Fisher test was used for comparison between groups. Repeated measurement data with normal distribution was compared using repeated measures ANOVA, whereas repeated measurement data with non-normal distribution was compared using generalized estimating equations. Bonferroni correction was used for multiple comparisons, and *P* < 0.05 indicated a statistically significant difference.

## Results

**Fig. 1:**
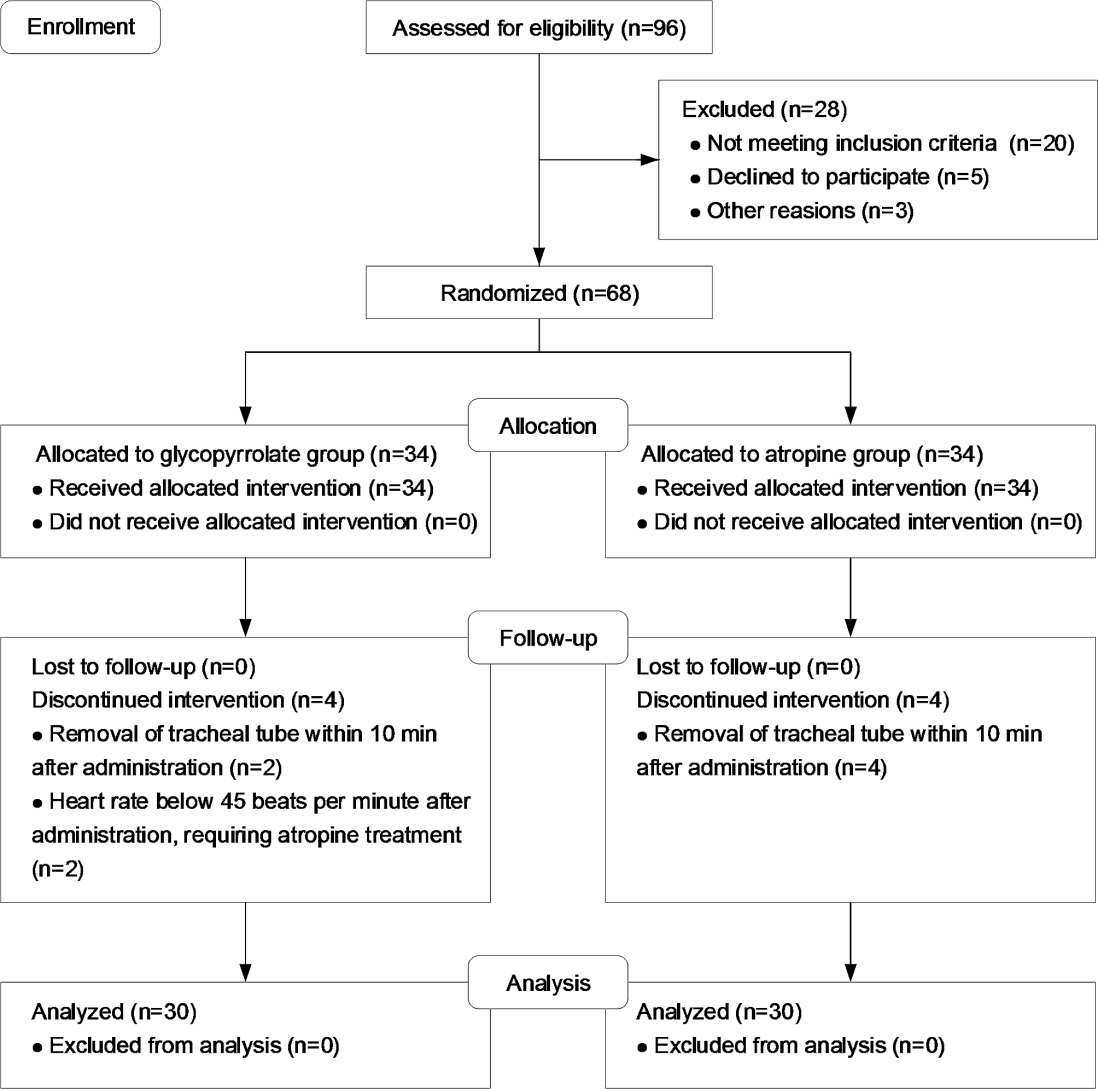
Flow diagram of study

A total of 96 patients were evaluated for eligibility, and 28 patients were excluded before randomization. As a result, 68 patients were included in this study who were equally randomized into 2 groups: group G (*n* = 34) and group A (*n* = 34). Two patients in group G and four in group A were excluded because their tracheal tubes were removed within 10 min after administration. Furthermore, two patients in group G were excluded because they received atropine as a remedy. 60 patients (30 in group G and 30 in group A) were included in the final analysis. The study procedure is shown in Fig. 1.

The two groups were similar in terms of gender, age, BMI, ASA class, surgery duration, anesthesia duration, and cisatracurium dose (Table 1).

**Table 1:** Patient characteristics and intraoperative data

Variable	Group G (n = 30)	Group A (n = 30)	*P* value
Age (yr)	69.0 (66.0–73.5)	67.0 (66.0–69.3)	0.328
Sex (M/F)	16/14	15/15	0.796
BMI (kg/m^2^)	24.4 ± 2.4	23.6 ± 3.3	0.314
ASA class (I/II)	11/19	13/17	0.598
Surgery duration (min)	89.5 (48.8–148.5)	83.5 (46.8–110.0)	0.779
Anesthesia duration (min)	101.0 (64.0–185.3)	104.0 (65.3–141.3)	0.824
Cisatracurium dose (mg/kg)	0.20 (0.17–0.30)	0.21 (0.19–0.25)	0.579

Data are expressed by median (interquartile range), number of patients, or mean ± standard deviation. Group G, glycopyrrolate group; Group A, atropine group; BMI, body mass index; ASA, American Society of Anesthesiologists.

**Fig. 2:**
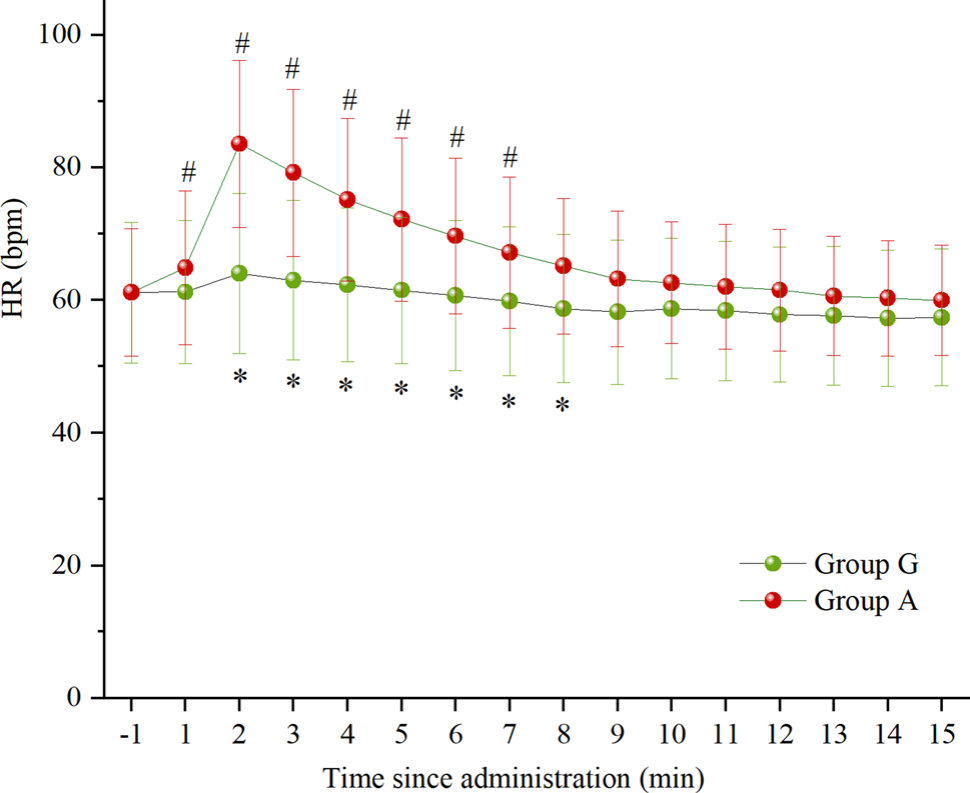
Heart rate at various times after administration

Data are expressed as mean ± standard deviation. *P < 0.05 vs. Group A, ^*#*^*P* < 0.05 vs. the baseline. HR, heart rate; Group G, glycopyrrolate group; Group A, atropine group.

HR was significantly lower in group G comepared to group A at 2, 3, 4, 5, 6, 7, and 8 min after administration (*P* < 0.001; *P* < 0.001; *P* < 0.001; *P* = 0.001; *P* = 0.004; *P* = 0.015; *P* = 0.024, respectively). Compared to the baseline, there was no statistically significant difference in HR at all time points after administration in group G (*P* > 0.05), whereas HR was significantly higher in group A at 1, 2, 3, 4, 5, 6, and 7 min after administration (*P* = 0.035; *P* < 0.001; *P* < 0.001; *P* < 0.001; *P* < 0.001; *P* < 0.001; *P* = 0.007, respectively). (Fig. 2)

**Fig. 3:**
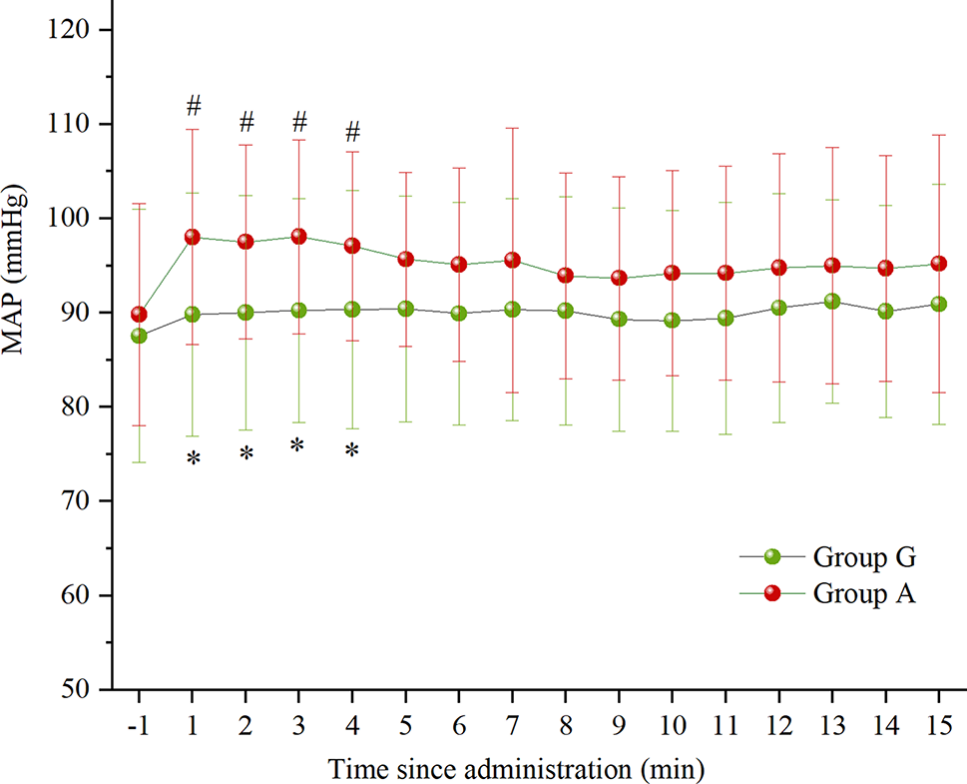
Mean arterial pressure at various times after administration

Data are expressed as mean ± standard deviation. *P < 0.05 vs. Group A, ^*#*^*P* < 0.05 vs. the baseline. MAP, mean arterial pressure; Group G, glycopyrrolate group; Group A, atropine group.

MAP was significantly lower in group G comepared to group A at 1, 2, 3, and 4 min after administration (*P* = 0.011; *P* = 0.014; *P* = 0.008; *P* = 0.026, respectively).

Compared with the baseline, there was no statistically significant difference in MAP at all time points after administration in group G (*P* > 0.05), whereas MAP was significantly higher in group A at 1, 2, 3, and 4 min after administration (*P* < 0.001; *P* < 0.001; *P* < 0.001; *P* = 0.001, respectively). (Fig. 3)

**Fig. 4:**
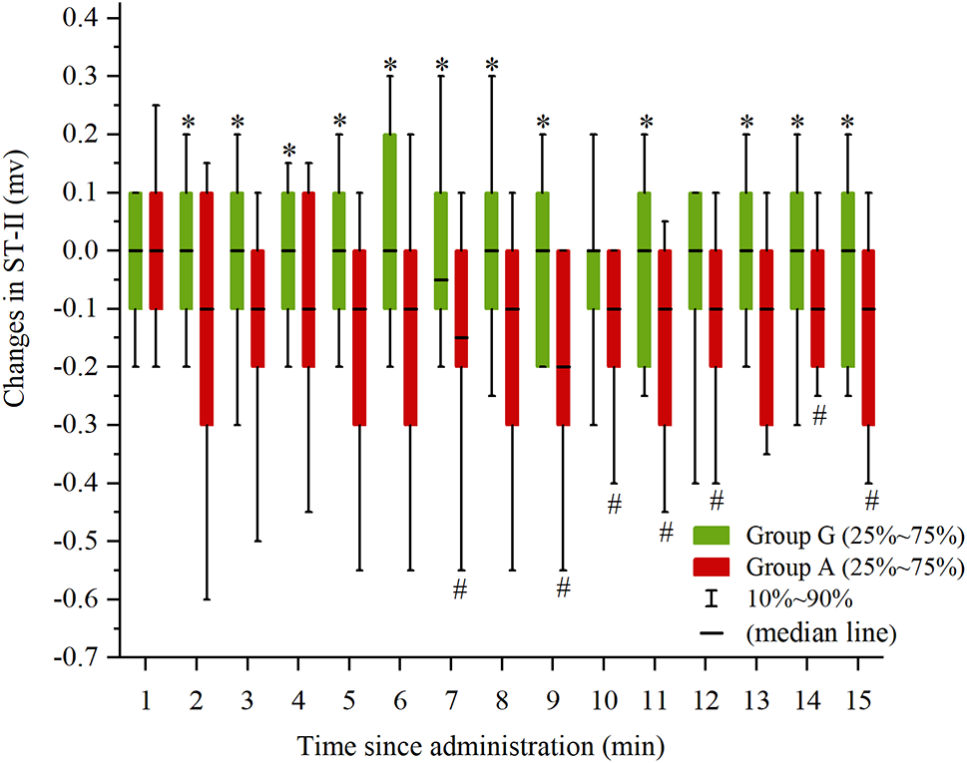
ST segment changes in lead II at various times after administration

Box = interquartile range; whiskers = 10th–90th percentile; median line = median. *P < 0.05 vs. Group A, ^*#*^*P* < 0.05 vs. the baseline. Changes in ST-II, ST segment changes in lead II; Group G, glycopyrrolate group; Group A, atropine group.

ST-II was significantly depressed in group A compared to group G at 2, 3, 4, 5, 6, 7, 8, 9, 11, 13, 14 and 15 min after administration (*P* = 0.017; *P* = 0.042; *P* = 0.040; *P* = 0.023; *P* = 0.010; *P* = 0.001; *P* = 0.005; *P* < 0.001; *P* = 0.004; *P* = 0.037; *P* = 0.025; *P* = 0.028, respectively). Compared to the baseline, ST-II changes in group G at each time point after administration showed no statistical significance (*P* > 0.05), whereas ST-II was significantly depressed in group A at 7, 9, 10, 11, 12, 14, and 15 min after administration (*P* = 0.032, *P* < 0.001, *P* = 0.004, *P* = 0.010, *P* = 0.033, *P* = 0.001, *P* = 0.047, respectively). (Fig. 4)

## Discussion

In this randomized controlled trial of elderly patients undergoing general anesthesia, G/N for reversing residual NMB had a more stable HR, MAP, and ST-II within 15 min after administration than A/N. In addition, the current study also discovered ST segment depression in lead II in elderly patients within 15 min after A/N administration, which was not observed after G/N administration.

Several studies have demonstrated that 20–40 ug/kg neostigmine can effectively reverse cisatracurium-induced shallow neuromuscular blockade [20–24]. Given the cardiovascular side effects of anticholinergic drugs in combination with neostigmine, dose selection in elderly patients should be cautious. The dose of neostigmine used in this trial was 20 ug/kg. Our findings showed that the HR of the elderly in the atropine group peaked 2 min after administration and then gradually slowed down. In contrast, the HR of the glycopyrrolate group did not change significantly. In an earlier multi-center study, Cozanitis et al. compared the effects of 10 ug/kg glycopyrrolate and 20 ug/kg atropine in combination with 50 ug/kg neostigmine in non-elderly patients. This study found that the change in HR of the glycopyrrolate group was significantly less than that of the atropine group and that the difference in HR between the two groups lasted up to 40 min after administration [18]. Similarly, an earlier study by Mirakhur et al. demonstrated better HR stability with 10 ug/kg glycopyrrolate in combination with 50 ug/kg neostigmine for reversal of NMB in elderly patients compared to 20 ug/kg atropine [25]. Our findings were largely consistent with these older evaluations, but some slight differences remained. First, unlike the study by Cozanitis et al., the difference in HR between the two groups in the current study lasted only up to 8 min after administration. This suggested that the lower the dose of antagonist, the smaller the effect on HR, and the shorter the duration of the difference in HR between the two groups. Furthermore, the atropine group experienced a greater initial increase in HR in this study, for which there were two possible explanations. On the one hand, this was due to the higher dose ratio of A/N (1:2) in our study. On the other hand, elderly patients may be more sensitive to atropine because their vagal tone is lower, resulting in a greater initial increase in HR.

In the present study, although the blood pressure changes in both groups were within the clinically acceptable range, the blood pressure fluctuations in elderly patients in the atropine group were significantly higher than those in the glycopyrrolate group. Furthermore, ST segment depression in lead II was observed at multiple time points following A/N administration, and ST segment still had not returned to baseline within 15 min. The ST depression may be a sign of coronary ischemia [26, 27], which could be associated with the atropine-induced increase in HR [28, 29]. However, there was no significant depression in ST-II in the glycopyrrolate group, implying that low-dose G/N may not affect myocardial perfusion in elderly patients. This would be especially beneficial for the elderly with underlying myocardial ischemia or without a clinically confirmed diagnosis of coronary artery disease.

A large retrospective study found no significant correlation between G/N for NMB and the occurrence of cardiovascular complications 30 days postoperatively, but its exploratory analysis identified an elderly population susceptible to the cardiovascular side effects of G/N administration [30]. In the current study, two patients experienced a transient initial tachycardia (> 100 bpm) following A/N, for which we gave only close observation without treatment. In contrast, two patients in the glycopyrrolate group had severe bradycardia (< 45 bpm). There have been a few case reports of bradycardia and atrio-ventricular block following G/N [31, 32]. This may be a reminder to be alert to the occurrence of bradycardia with G/N in elderly patients.

There are several limitations to this study. Firstly, for the baseline, we only collected data 1 min before administration, and it would be more rigorous and convincing to average data over a period of time. Secondly, for ischemia detection, only ST segment in lead II was monitored using a 3-lead system, whereas a 5-lead system with a lateral ventricular lead should be a better choice, more sensitive than lead II and less intimidating than a 12 lead.

In conclusion, G/N for reversing residual NMB in the elderly has a more stable HR, MAP, and ST-II within 15 min after administration than A/N. Furthermore, we find that ST segment depression is significant in elderly patients within 15 min after A/N administration, which may increase the risk of perioperative myocardial ischemia.

## Data Availability

The datasets used and analyzed during the current study are available from the corresponding author on reasonable request.

## Abbreviations

G/N: glycopyrrolate-neostigmine
A/N: atropine-neostigmine
NMB: neuromuscular blockade
HR: heart rate
MAP: mean arterial pressure
ST-II: ST segment in lead II
Changes in ST-II: ST segment changes in lead II
CONSORT: Consolidated Standards of Reporting Trials
ASA: American Society of Anesthesiology
BMI: body mass index
NBP: noninvasive blood pressure
ECG: electrocardiogram
Sp0_2_: pulse oxygen saturation
BIS: bispectral index
